# Serial MRI documentation of sequestrated lumbar disc resorption during conservative management: a case report

**DOI:** 10.3389/fpain.2026.1853632

**Published:** 2026-09-03

**Authors:** Zhou Zhang, Zhiliang Yang, Yongqi Zhao, Yuchen Guo, Guangwen Wu

**Affiliations:** 1 School of Orthopedics and Traumatology, Fujian University of Traditional Chinese Medicine, Fuzhou, China; 2 Key Laboratory of Orthopedics & Traumatology of Traditional Chinese Medicine and Rehabilitation, Ministry of Education, Fujian University of Traditional Chinese Medicine, Fuzhou, China

**Keywords:** acupuncture, case report, lumbar disc herniation, sequestrated disc, serial MRI, spontaneous resorption, traditional Chinese medicine

## Abstract

**Background:**

Although spontaneous resorption in lumbar disc herniation (LDH) is a recognized phenomenon, systematic visual evidence of its temporal progression remains limited. This report utilizes short-interval serial magnetic resonance imaging (MRI) to document the longitudinal clinico-radiological evolution of a sequestrated LDH managed within an integrative conservative framework, providing a continuous imaging-clinical timeline linking structural resolution to symptomatic recovery.

**Case presentation:**

A 33-year-old man presented with acute-onset low back pain and right lower limb radicular pain. MRI demonstrated a right posterolateral L4/5 disc extrusion with sequestration, featuring peripheral contrast enhancement (the “bull's-eye” sign). After declining non-steroidal anti-inflammatory drugs (NSAIDs), the patient opted for a 16-week integrative regimen comprising acupuncture and modified Duhuo Jisheng decoction. Serial MRI across five time points enabled high-resolution longitudinal tracking of morphological changes. Imaging documented the complete resorption of the free sequestrated fragment, achieving a 79.3% reduction in the herniation ratio alongside a 77.7% reduction in the cross-sectional area at 4 months. These morphological adjustments coincided with substantial clinical recovery, with the Visual Analogue Scale (VAS) score decreasing from 8 to 0 and the Japanese Orthopaedic Association (JOA) score improving from 5 to 28. A 5-month post-treatment follow-up confirmed sustained resolution without recurrence, showing only residual L4/5 disc bulging.

**Conclusions:**

Serial MRI dynamically captured the progressive resorption of a sequestrated L4/5 disc and its concordant clinical recovery during integrative conservative management. While this approach was temporally associated with favorable outcomes, the observational design precludes definitive causal attribution. This report suggests that such management may provide a supportive clinical window allowing spontaneous resorption to proceed; however, higher-level controlled studies are required to differentiate potential therapeutic modulation from the natural history of disc regression.

## 1 Introduction

Lumbar disc herniation (LDH) is a prevalent cause of low back pain and radicular symptoms (1, 2). Sequestrated herniations frequently undergo spontaneous resorption via inflammatory and immune-mediated mechanisms (3). However, the radiological trajectory of disc regression under conservative care remains poorly documented, as most reports rely solely on baseline and endpoint imaging (4–6). This report utilizes short-interval serial MRI to provide high-resolution temporal documentation of structural regression and symptomatic recovery in a sequestrated L4/5 LDH managed with an integrative conservative approach.

## 2 Case presentation

### 2.1 History

On June 16, 2025, a 33-year-old sedentary worker presented with a 1-day history of acute low back pain radiating to the right calf. His occupation involved prolonged sitting and frequent driving. He denied any history of trauma, heavy lifting, or prior episodes of significant low back or leg pain. The onset of symptoms was sudden, beginning with localized low back pain, followed by severe radicular pain along the lateral aspect of the right lower extremity, which markedly limited his activities of daily living. There were no associated bowel or bladder disturbances, perineal numbness, or progressive lower extremity weakness to suggest cauda equina syndrome. He also did not experience fever, nocturnal pain, or unexplained weight loss. He had previously been in good health, with no history of chronic illness or regular medication use.

### 2.2 Diagnostic assessment

On physical examination, lumbar mobility was significantly restricted, with flexion limited to approximately 10° and near-complete loss of extension, while physiological lordosis was preserved. Lumbar lateral flexion and rotation were reduced and asymmetric, measuring approximately 15° on the left and 5° on the right. Tenderness was found over the right paravertebral area adjacent to the L5 spinous process, and percussion over L3–5 spinous processes elicited pain. Neurological examination showed that muscle strength in the right lower limb (Medical Research Council grade 5/5) and muscle tone in all limbs were normal. Deep tendon reflexes, including the patellar and Achilles reflexes, were symmetric, and plantar responses were flexor bilaterally. Sensory testing revealed mild hypoesthesia over the lateral aspect of the right calf and the dorsomedial aspect of the right foot, consistent with a right L5 dermatomal distribution. Provocative testing yielded a positive right straight leg raise (SLR) test at 30°, which reproduced the radicular pain, while a crossed SLR test on the left was positive at 45°, further exacerbating the contralateral right-sided symptoms.

**Figure 1:**
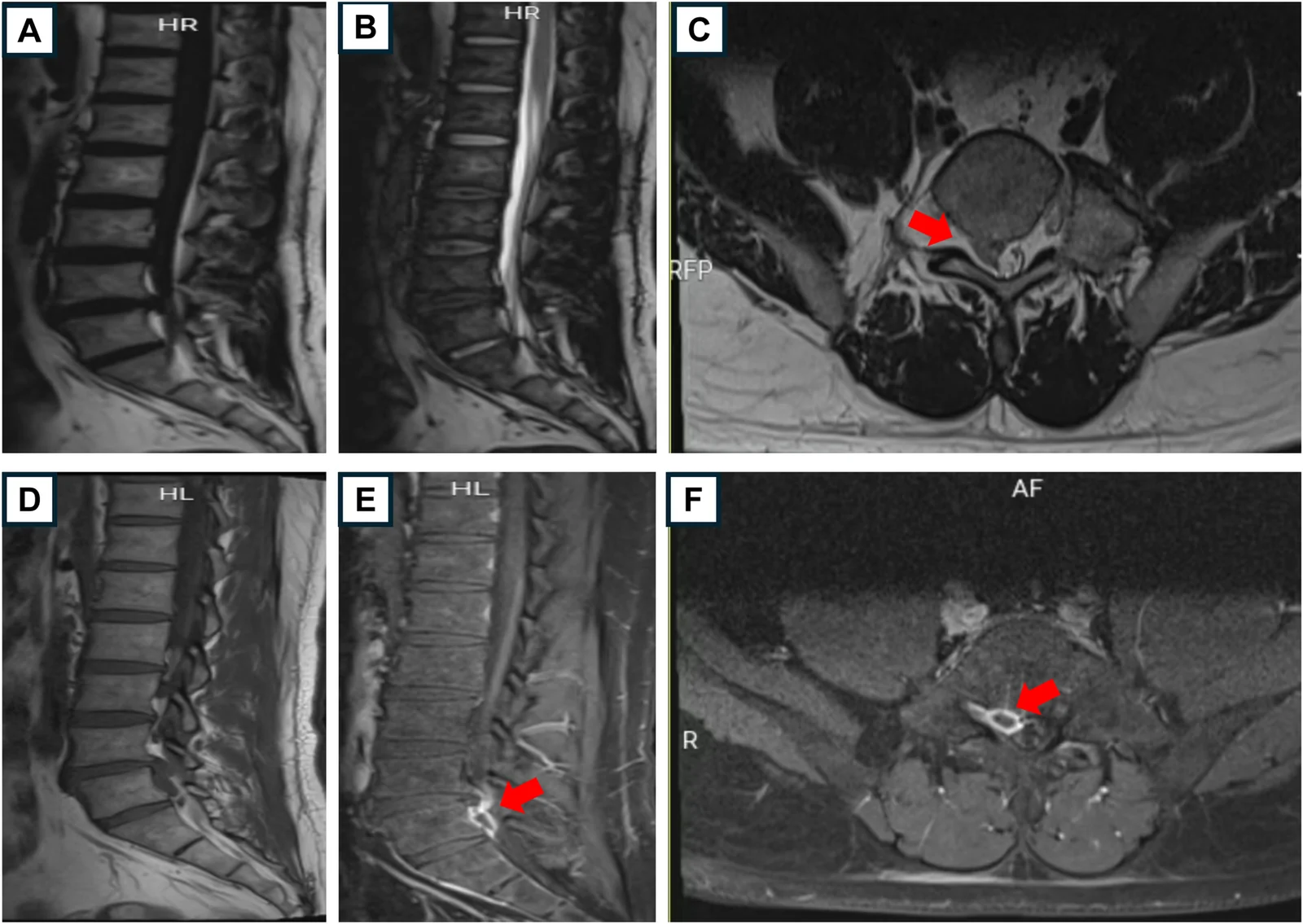
Initial lumbar MRI findings. Non-contrast MRI (June 16, 2025) and contrast-enhanced MRI (June 19, 2025) demonstrate an L4/5 disc extrusion with sequestration. **(A–C)** Axial non-contrast images show a herniated nucleus pulposus occupying 40.6% of the spinal canal (red arrow). **(D,E)** Sagittal contrast-enhanced images indicate partial sequestration at the L4/5 level (red arrow). **(F)** Axial contrast-enhanced image reveals peripheral rim enhancement surrounding the sequestrated disc fragment, a classic radiographic phenomenon referred to as the “bull's-eye sign” (red arrow).

On initial assessment, the patient reported a Visual Analogue Scale (VAS) score of 8 and a Japanese Orthopaedic Association (JOA) score of 5. The MRI demonstrated a large right posterolateral disc extrusion at the L4/5 level that appeared to compress the dural sac and the right L5 nerve root. The herniation ratio was 83.1%. The greatest cross-sectional area of the spinal canal was 3.2 cm^2^, and that of the herniated fragment was 1.3 cm^2^, accounting for 40.6% of the area (Figures 1A–C). Three days after the plain MRI, lumbar contrast-enhanced MRI was performed to further characterize the herniated disc. The enhanced images confirmed a typical bull's-eye sign indicative of a sequestrated disc fragment (Figures 1D–F). The clinical presentation and MRI features were suggestive of a sequestrated right L4/5 LDH with associated right L5 radiculopathy.

All radiological measurements were performed using PACS-integrated measurement tools by two experienced radiologists independently, who were blinded to the clinical data. The herniation ratio was calculated as (a – b)/a   ×   100%, where ‘a’ is the anteroposterior diameter of the spinal canal and ‘b’ is the distance from the apex of the herniated mass to the posterior wall of the spinal canal on axial T2-weighted images. The cross-sectional area of the herniated mass was determined by manual tracing of the fragment margins at the level of maximal canal compromise, explicitly including the peripheral enhancing rim when present on contrast-enhanced images. Each parameter was measured three times by each observer in separate sessions, and the mean values were used for analysis. Inter-observer reliability, assessed via the intraclass correlation coefficient, demonstrated excellent agreement (ICC > 0.90 for all parameters).

### 2.3 Therapeutic intervention

The patient received a 16-week integrative conservative regimen comprising acupuncture and oral TCM. The acupuncture protocol was formulated in accordance with traditional meridian theory. The overarching therapeutic strategy primarily targeted the Bladder and Gallbladder meridians—adhering to the classical principle “wherever a meridian passes, its indications follow"—and employed a distal-proximal pairing strategy alongside the “He-Sea treatment for internal organs” principle to address both the root cause and clinical manifestations. Guided by these principles, the following acupoints were selected: bilateral Shenshu (BL23), Qihaishu (BL24), Dachangshu (BL25), Guanyuanshu (BL26), Xiaochangshu (BL27), and Baihuanshu (BL30) to tonify kidney qi and provide targeted L2–S2 segmental neuromodulation; ipsilateral Huantiao (GB30) and Weizhong (BL40) to manage sciatica and lower limb radicular pain; and lumbosacral Ashi points to alleviate regional myofascial dysfunction. In the prone position, the patient's skin was disinfected with 75% ethanol. Disposable sterile filiform needles (0.4 mm   ×   60 mm; Yunlong, Jiangsu, China) were inserted perpendicularly or obliquely to a safe depth of 25–35 mm, targeting the periosteum adjacent to the lumbar vertebrae. Following the elicitation of de qi (a soreness-distention-pressure sensation), twisting, rotating, and lifting-thrusting manipulations were performed using the ping bu ping xie (even reinforcement and reduction) technique. Needles were retained for 30 min per session, with manual stimulation applied every 10 min. Treatment was administered weekly for four consecutive 4-week courses, totaling 16 weeks.

Concurrently, the patient was prescribed a modified Duhuo Jisheng Decoction (DHJSD). This prescription was formulated based on syndrome differentiation (*bian zheng lun zhi)* for ‘Bi syndrome’ (*yao tui tong*) characterized by qi stagnation and blood stasis with underlying liver-kidney deficiency, adhering to the therapeutic principle of activating blood circulation, resolving stasis, unblocking collaterals, and tonifying the liver and kidney. The formula comprised *Chenpi* (6 g), *Muxiang* (9 g), *Gancao* (6 g), *Mudanpi* (9 g), *Shenjincao* (12 g), *Sangjisheng* (15 g), *Taoren* (9 g), *Yanhusuo* (15 g), *Niuxi* (15 g), *Duzhong* (10 g), *Dangshen* (9 g), *Fuling* (15 g), *Shanyao* (15 g), and *Zexie* (12 g). The decoction was prepared by boiling the herbs in 500 mL of water until the volume was reduced to 300 mL. It was administered orally in two divided doses daily for 16 consecutive weeks, with no interim modifications to the prescription.

Surgical intervention, including microdiscectomy, was thoroughly discussed with the patient at the initial consultation given the MRI-confirmed sequestration with nerve root compression. However, after evaluation by a consulting orthopedic surgeon, it was determined that the absence of progressive neurological deficits, cauda equina syndrome, or intractable pain rendered conservative management a reasonable alternative. After being fully informed of the risks, benefits, and expected recovery trajectories, the patient elected to pursue a trial of non-operative care. A shared decision was made to proceed with an integrative TCM-based approach under close clinical and radiological surveillance, with a clear contingency plan for surgical referral should neurological deterioration occur or symptoms fail to improve.

**Figure 2:**
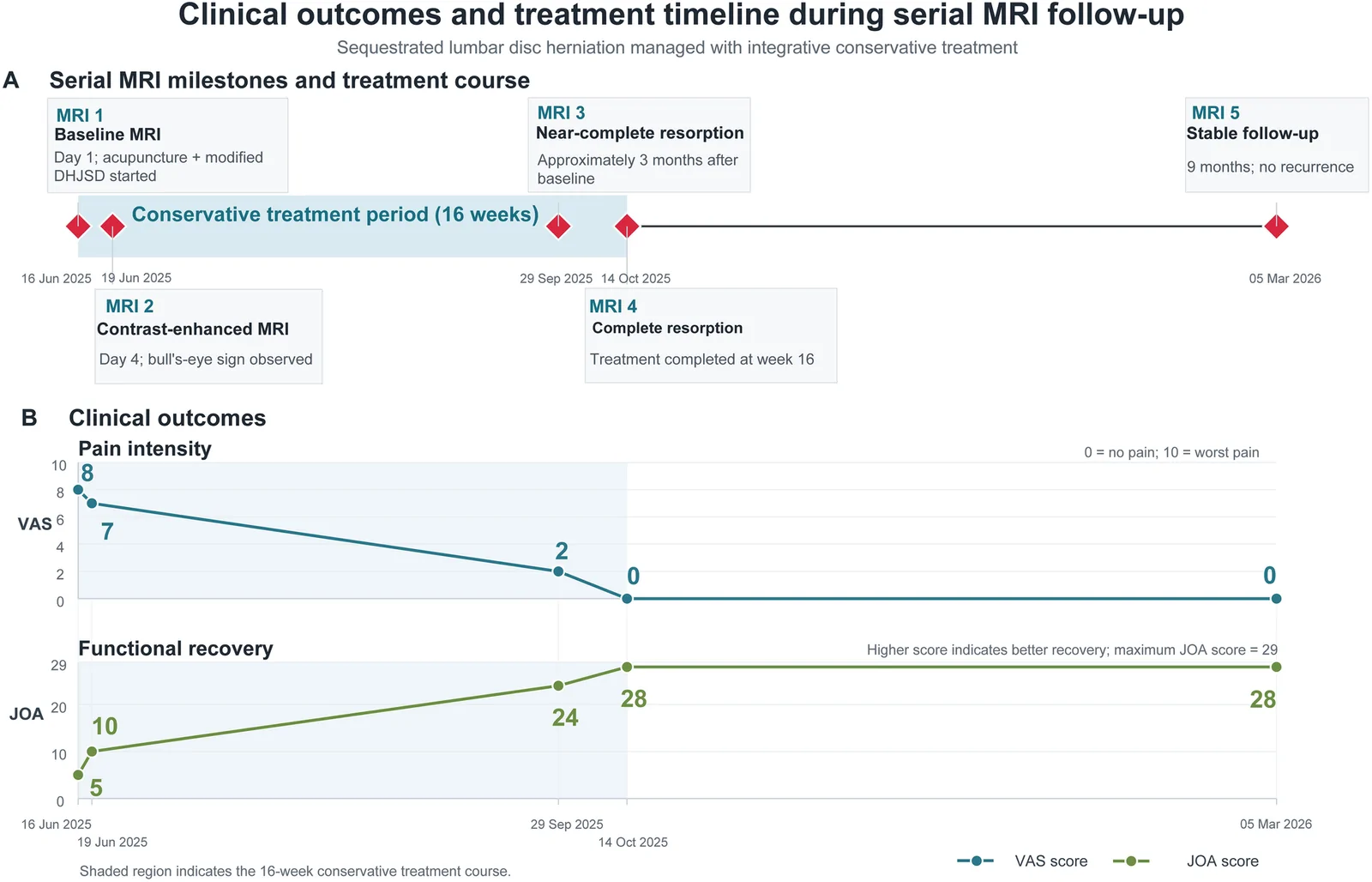
Clinical outcomes and treatment timeline. **(A)** Serial MRI milestones and treatment course from baseline to the 9-month follow-up. The shaded band indicates the 16-week conservative treatment period, during which the patient received weekly acupuncture and oral modified Duhuo Jisheng decoction. MRI assessments were performed at baseline (June 16, 2025), Day 4 with contrast enhancement (June 19, 2025), approximately 3 months (September 29, 2025), 4 months at treatment completion (October 14, 2025), and 9 months (March 5, 2026). **(B)** Longitudinal changes in pain and functional recovery. The Visual Analogue Scale (VAS) score decreased from 8 to 0, while the Japanese Orthopaedic Association (JOA) score improved from 5 to 28 and remained stable at follow-up.

As part of this conservative regimen, the patient was advised to undertake relative rest and to avoid prolonged sitting and driving to reduce lumbosacral strain. He adhered strictly to these recommendations and reported no treatment-related adverse events. The overall treatment course, clinical characteristics, and serial MRI assessments across different time points are summarized in Table 1 and Figure 2A.

**Table 1:** Clinical characteristics, imaging findings, and functional outcomes across different time points.

Time Point	Treatment period	Intervention	VAS	JOA Score	SLR (Right)	Neurological Examination	MRI Findings	Herniation Ratio (%)	Maximum Cross-sectional Area of Herniated Mass (cm^2^)	Institution	MRI Field Strength
June 16, 2025	Day 1 (Baseline)	Acupuncture + TCM	8	5	30° (+)	Hypoesthesia: lateral calf, medial dorsal foot	L4/5 disc herniation with right nerve root compression	83.1	1.3	Changxing County Hospital of TCM	1.5-T
June 19, 2025	Day 4	Acupuncture + TCM	7	10	35° (+)	Hypoesthesia: lateral calf, medial dorsal foot	L4/5 extrusion with sequestration, right root compression (bull's-eye sign)	89.6	1.4	Changxing County Hospital of TCM	1.5-T
September 29, 2025	approximately 3 months	Acupuncture + TCM	2	24	75° (+)	Hypoesthesia: lateral calf; Medial dorsal foot: normal	Sequestration: near-complete resorption; Extrusion: partial resorption	19.5	0.37	South Taihu Hospital of Huzhou	3.0-T
October 14, 2025	approximately 4 months	Treatment completed	0	28	90° (−)	Normal	Sequestration: complete resorption; Residual L4/5 disc bulging	17.2	0.29	Huzhou Central Hospital	3.0-T
March 5, 2026	approximately 9 months (Follow-up)	None (Follow-up window)	0	28	90° (−)	Normal	Sequestration: complete resorption; Residual L4/5 disc bulging	17.2	0.29	Changxing County Hospital of TCM	1.5-T

SLR, straight leg raise; VAS, visual analogue scale; JOA, Japanese orthopaedic association; MRI, magnetic resonance imaging. For SLR test results, (+) indicates a positive test with radicular pain, and (–) indicates a negative test without radicular symptoms. VAS scores range from 0 to 10 (higher scores indicate more severe pain). JOA scores range from 0 to 29 (higher scores indicate better functional status). The herniation ratio was calculated as follows: Herniation Ratio = (a - b)/a   ×   100%, where ‘a’ represents the anteroposterior diameter of the spinal canal and ‘b’ represents the distance from the apex of the herniated mass to the posterior wall of the spinal canal. The 4-month longitudinal reduction in the herniation ratio was calculated as: (Baseline ratio - 4-month ratio)/Baseline ratio   ×   100%, yielding 79.3%. The area-based resorption rate was calculated based on the maximum cross-sectional area as: (Baseline area - 4-month area)/Baseline area   ×   100%, yielding 77.7%. All measurements were independently verified by two experienced radiologists who were blinded to the clinical data.

### 2.4 Response to treatment

**Figure 3:**
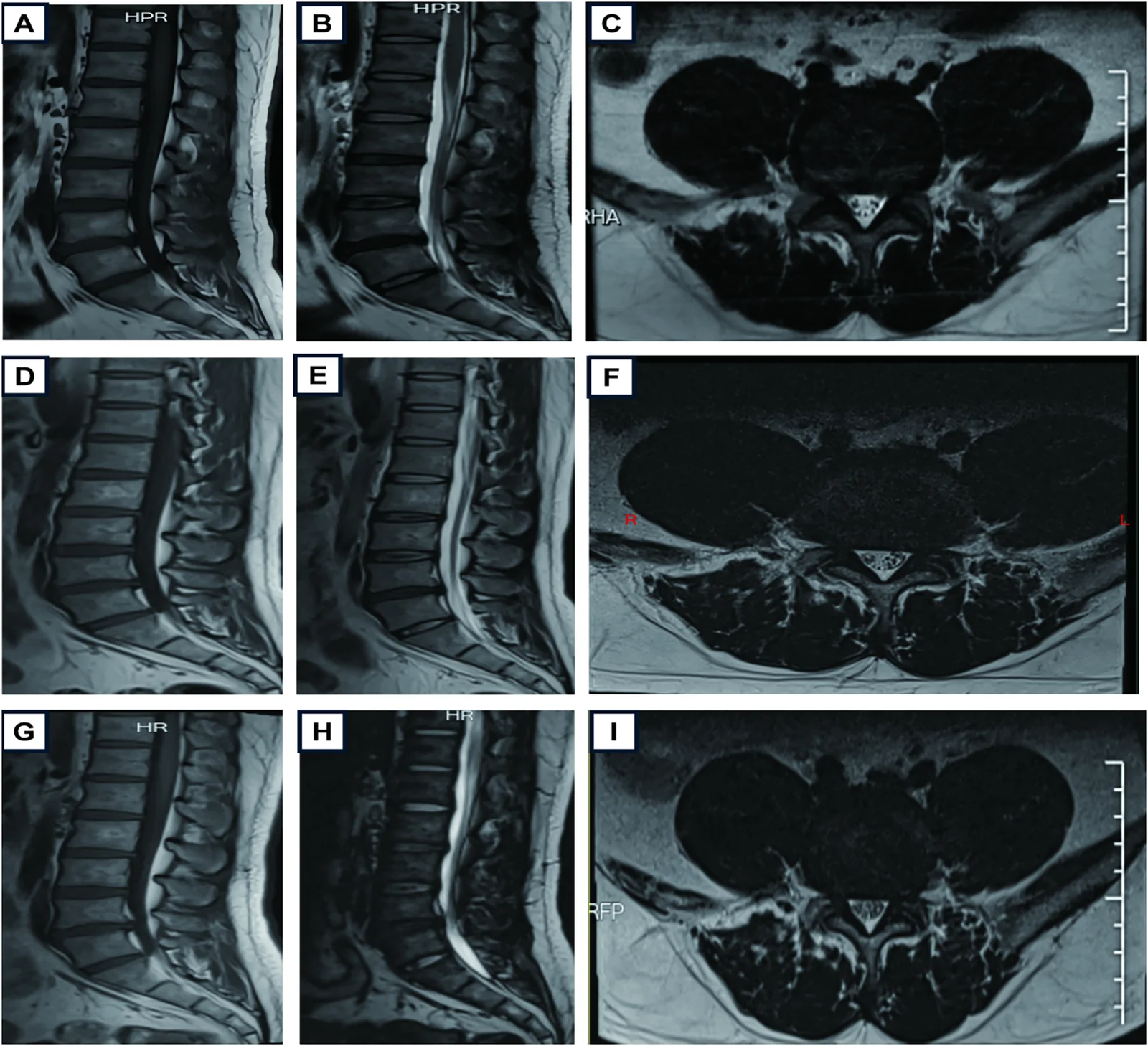
Serial follow-up lumbar MRI. **(A–C)** At approximately 3 months post-baseline (September 29, 2025), non-contrast MRI reveals near-complete resorption of the sequestrated nucleus pulposus at the L4/5 level. **(D–F)** At the 4-month follow-up (October 14, 2025), non-contrast MRI demonstrates complete resorption of the free sequestrated fragment alongside marked regression of the extruded component, leaving only a persistent residual disc bulge (herniation ratio: 17.2%; area-based resorption rate: 77.7%). **(G–I)** At the 9-month longitudinal follow-up (March 5, 2026; 5 months after treatment cessation), MRI shows a stable, persistent L4/5 disc bulge without evidence of recurrence or renewed nerve root compression.

After approximately three months of conservative treatment, the patient reported significant improvement in low back pain and right-sided radicular symptoms, alongside a restoration of normal ambulation and nearly complete recovery of activities of daily living. Pain scores demonstrated substantial improvement, with the VAS decreasing from 8 to 2 and the JOA score increasing from 5 to 24. Lumbar range of motion had improved, with flexion measuring approximately 90°, extension 15°, and bilateral lateral flexion and rotation 30°. Paraspinal tenderness at L3-L5 was no longer present. The right SLR test transitioned to negative, eliciting no radicular symptoms until exceeding 75°. Muscle strength in both lower extremities was normal (MRC 5/5). Sensory deficits in the right lower limb had markedly improved, with only minimal residual hypoesthesia in the lateral calf, while sensation in the right foot had returned to normal. Deep tendon reflexes (patellar and Achilles) were symmetric and within normal limits. Follow-up lumbar MRI performed on September 29, 2025, using a high-resolution 3.0 T MRI scanner (South Taihu Hospital of Huzhou, Huzhou, China), demonstrated near-complete resorption of the L4/5 sequestrated disc fragment at the posterior margin of the L5 vertebral body. The imaging revealed no significant residual compression of the right nerve root. Quantitatively, the herniation ratio had decreased to 19.5%, and the residual herniated fragment measured 0.37 cm^2^ (Figures 3A–C).

At the end of the 16-week treatment course (October 14, 2025), the patient underwent outpatient evaluation. Lumbar range of motion was within normal limits, with flexion approximately 90°, extension 20°, lateral flexion 30°, and bilateral rotation up to 30°. The straight leg raise test was negative at 90° bilaterally. Sensory function in the right lower extremity was completely restored. Deep tendon reflexes were symmetric, and the Babinski sign was negative bilaterally. Correspondingly, the VAS score was reduced to 0, and the JOA score improved to 28 (Figure 2B). Concurrently, a repeat lumbar MRI was performed using a high-resolution 3.0-T system (Huzhou Central Hospital, Huzhou, China). The imaging demonstrated complete resorption of the sequestrated fragment along with marked regression of the extruded component, leaving only a stable, residual disc bulge at the L4/5 level. Quantitatively, the herniation ratio decreased from the baseline of 83.1% to 17.2%, representing a 79.3% relative reduction. Furthermore, the cross-sectional area of the herniated mass was reduced to 0.29 cm^2^, yielding an area-based resorption rate of 77.7% (Figures 3D–F).

On March 5, 2026 (9 months post-baseline), the patient reported sustained complete resolution of all symptoms. Clinical assessments remained excellent (VAS 0, JOA 28), with fully preserved lumbar mobility, negative SLR, and normal neurological findings. A follow-up MRI confirmed stable residual L4/5 disc bulging with no recurrence of sequestration or nerve root compression (Figures 3G–I).

## 3 Discussion

The spontaneous resorption of sequestrated LDH is well-documented, with reported resorption rates of 87%–96% for sequestrated discs versus 37%–41% for protruded discs (7–9). The present case contributes several specific features beyond standard reporting: short-interval serial MRI at five time points capturing the acute inflammatory phase, synchronous clinical-imaging correlation at each time point, and an extended 5-month post-treatment follow-up confirming sustained resolution. Quantitatively, a 79.3% reduction in herniation ratio and a 77.7% reduction in cross-sectional area were documented over 4 months. However, spontaneous regression of sequestrated fragments is a recognized natural phenomenon, and this single observational case cannot establish causality between the administered interventions and the observed morphological changes.

Biologically, LDH resorption is driven by inflammation, neovascularization, macrophage infiltration, and matrix metalloproteinase-mediated ECM degradation (10–12). The substantially higher resorption rates observed in sequestrated versus contained herniations are attributable to differences in anatomical exposure: disruption of the annulus fibrosus—and often the posterior longitudinal ligament—exposes the previously immune-privileged nucleus pulposus to the epidural vasculature, triggering a robust autoimmune inflammatory cascade that accelerates phagocytosis and fragment clearance (13, 14). In contrast, contained herniations, in which the annulus remains intact, shield the nucleus pulposus from epidural exposure, thereby eliciting a substantially attenuated inflammatory response that accounts for their markedly lower resorption rates. The ‘bull's-eye sign’ on contrast-enhanced MRI—peripheral rim enhancement indicating active granulation and neovascularization—represents a recognized favorable prognostic marker for robust resorption (15). In the present case, the transient increase in herniated mass dimensions observed at Day 4 likely reflects this acute inflammatory edema rather than true structural progression or clinical deterioration. Given that local cellular and inflammatory markers were not directly quantified in this patient, the pathways described herein serve as a mechanistic framework to explain the observed morphological changes, rather than direct empirical evidence.

The severe radicular pain that accompanies disc resorption frequently drives premature surgical intervention. In this case, the integrative regimen—comprising acupuncture, modified DHJSD, and activity modification—was temporally associated with rapid symptomatic relief (VAS score decreased from 8 to 0; JOA score improved from 5 to 28). While current LDH guidelines classify TCM interventions as weakly recommended (16–18), accumulating clinical evidence suggests that acupuncture combined with DHJSD is associated with reductions in pain and disability scores, potentially through modulation of pro-inflammatory cytokines (IL-1β, IL-6, TNF-α) implicated in radicular hyperalgesia (19). Preclinical studies have further demonstrated that DHJSD constituents exhibit anti-inflammatory and chondroprotective properties in experimental models, while acupuncture has been shown to enhance regional microcirculation and modulate neuroimmune interactions at the spinal level. The traditional concept of ‘unblocking collaterals’ aligns conceptually with these observations, suggesting that enhanced perfusion may support metabolite clearance and tissue remodeling (20). However, whether these interventions exert any direct effect on the resorption process cannot be determined from the present data, and the observed clinical course may reflect the natural history of pain resolution coinciding with spontaneous fragment regression. The clinical value of the integrative approach in this case therefore lies in its capacity to manage acute symptoms effectively, thereby preserving a safe window during which natural resorption could proceed.

The ‘inflammation paradox’ merits consideration: localized inflammatory cascades are requisite for macrophage-driven fragment clearance, yet excessive neuroinflammation drives severe radicular pain. Current insights suggest that integrative TCM strategies may exert a targeted immunomodulatory rather than immunosuppressive effect—potentially mitigating pain-inducing inflammation at the disc-nerve root interface while permitting macrophage-mediated fragment clearance to proceed. This hypothesis, while biologically plausible, requires validation through controlled studies incorporating direct biomarker measurements.

Several limitations should be considered. First, as a single observational case without a control group, definitive causality between the conservative interventions and disc resorption cannot be established; spontaneous resorption is a well-documented natural phenomenon in sequestrated LDH. Consequently, the contribution of this report lies in the high-resolution temporal documentation of the resorption trajectory rather than in claims of therapeutic efficacy. Second, co-intervention bias is inherent: the patient received acupuncture, herbal medicine, and activity modification simultaneously, precluding the isolation of individual modality effects. Third, no pre-baseline imaging was available to capture the initial transition from extrusion to sequestration. Fourth, non-uniform imaging intervals between Day 4 and the 3-month follow-up left intermediate inflection points of the resorption trajectory unmapped. Future studies employing randomized controlled designs with standardized protocols, objective imaging endpoints, and direct biomarker measurements are warranted to differentiate potential therapeutic modulation from spontaneous resolution.

## 4 Conclusions

Serial MRI documented the concordant clinical recovery and radiological resorption of a sequestrated L4/5 LDH during integrative conservative management. Spontaneous resorption is a well-established aspect of the natural history of sequestrated fragments, and this single observational case cannot establish causality between the TCM intervention and the observed structural regression. For highly selected patients without progressive neurological deficits under rigorous monitoring, an integrative conservative approach may provide a safe clinical window permitting natural resorption to proceed. Controlled studies incorporating standardized imaging protocols and direct biomarker measurements are required to differentiate therapeutic modulation from spontaneous resolution.

### 4.1 Permission to reuse and copyright

Permission must be obtained for the use of copyrighted material from other sources (including the web).

## Data Availability

The raw data supporting the conclusions of this article will be made available by the authors, without undue reservation.
